# Cytochrome c oxidase dependent respiration is essential for T cell activation, proliferation and memory formation

**DOI:** 10.21203/rs.3.rs-4875322/v2

**Published:** 2024-09-16

**Authors:** Tatiana N. Tarasenko, Emily Warren, Amanda Fuchs, Bharati Singh, Jose Marin, Marten Szibor, Peter J. McGuire

**Affiliations:** 1 Metabolism, Infection, and Immunity Section, National Human Genome Research Institute, National Institutes of Health, Bethesda, MD 20892, USA.; 2 BioMediTech & Tampere University Hospital, Faculty of Medicine and Health Technology, 33014 Tampere University, Finland; 3 Department of Cardiothoracic Surgery, Center for Sepsis Control and Care (CSCC), Jena University Hospital, 07747 Jena, Germany

## Abstract

T cell activation, proliferation, and differentiation are fundamentally driven by shifts in cellular metabolism, with mitochondria playing a central role. Cytochrome c oxidase (COX, complex IV) is a key player in this process, as its activity is crucial for apoptosis, mtDNA maintenance, mitochondrial transcription, and mitochondrial respiration (MR), all of which influence T cell fate and function. Despite its known roles, the specific functions of COX required for T cell activity in vivo remain unclear. To isolate the role of MR in T cell function, we reintroduced this capability in COX-deficient T cells using an alternative oxidase (AOX) from *Ciona intestinalis*. Our findings demonstrate that MR is vital for maintaining metabolic balance during T cell activation by alleviating electron pressure from metabolic reprogramming and preserving redox homeostasis. We further showed that AOX mitigates apoptosis, prevents metabolic disruptions in glycolysis and the tricarboxylic acid cycle, and improves mtDNA maintenance and transcription, indicating that these disturbances are secondary to impaired MR in the absence of COX. Most importantly, the introduction of AOX restored robust effector and memory T cell generation and function in COX-deficient cells. These results highlight the essential role of COX-dependent MR in ensuring cellular health and underscore its pivotal role in T cell proliferation and differentiation.

## INTRODUCTION

Metabolic reprogramming during T cell activation and differentiation is critical to meet the bioenergetic and biosynthetic demands required for function^1, 2^. While glycolysis has been emphasized in immunometabolism studies, it is important to note that oxidative phosphorylation (OXPHOS) is also integral, with individual electron transport chain (ETC) complexes having unique roles. Seminal findings in genetic models of OXPHOS deficiency demonstrated the unique contributions of two of these ETC complexes. Coenzyme Q:cytochrome c oxidoreductase (complex III) was found to be involved in reactive oxygen species (ROS) production, which is essential for T cell activation^3^. Further work by our group revealed that cytochrome c oxidase (COX, complex IV) prevents cytochrome c-dependent apoptosis in T cells following activation, with deficiency leading to impaired differentiation and function, producing immunodeficiency^4^.

Although COX is involved in various processes such as apoptosis and mtDNA maintenance and transcription^4, 5^, we hypothesized that all COX-dependent effects in T cells fundamentally stem from its role in mitochondrial respiration (MR). COX-dependent MR is the process by which cells generate ATP through the ETC, whereby COX receives electrons from cytochrome c and transfers them to oxygen, producing water. The consumption of oxygen is a critical component of MR, as it ensures the continuation of the electron flow and the production of energy in the form of ATP. To directly test our hypothesis, we selectively re-established this one aspect of COX function using the alternative oxidase (AOX) from *Ciona intestinalis*. AOX is a 37 kDa mitochondrial protein that functions upstream of complex III, to sustain the electron transport chain and ATP production^6, 7, 8^. Similar to COX, AOX respires by transferring electrons from ubiquinol to molecular oxygen, reducing it to water. Unlike COX, the AOX is non-protonmotive, and thus relies upon complexes I and II for establishing a proton gradient in the intermembrane space. By incorporating AOX, we were able to isolate and scrutinize the individual contribution of MR to T cell activation, proliferation, and memory formation. AOX-mediated MR restored many aspects of T cell functions, including proliferation and differentiation, particularly in memory T cells. Our findings suggest that the fundamental role of MR via COX is integral for proper T cell activation and function.

## RESULTS

### MR restoration impacts cellular dynamics

**Fig 1.**
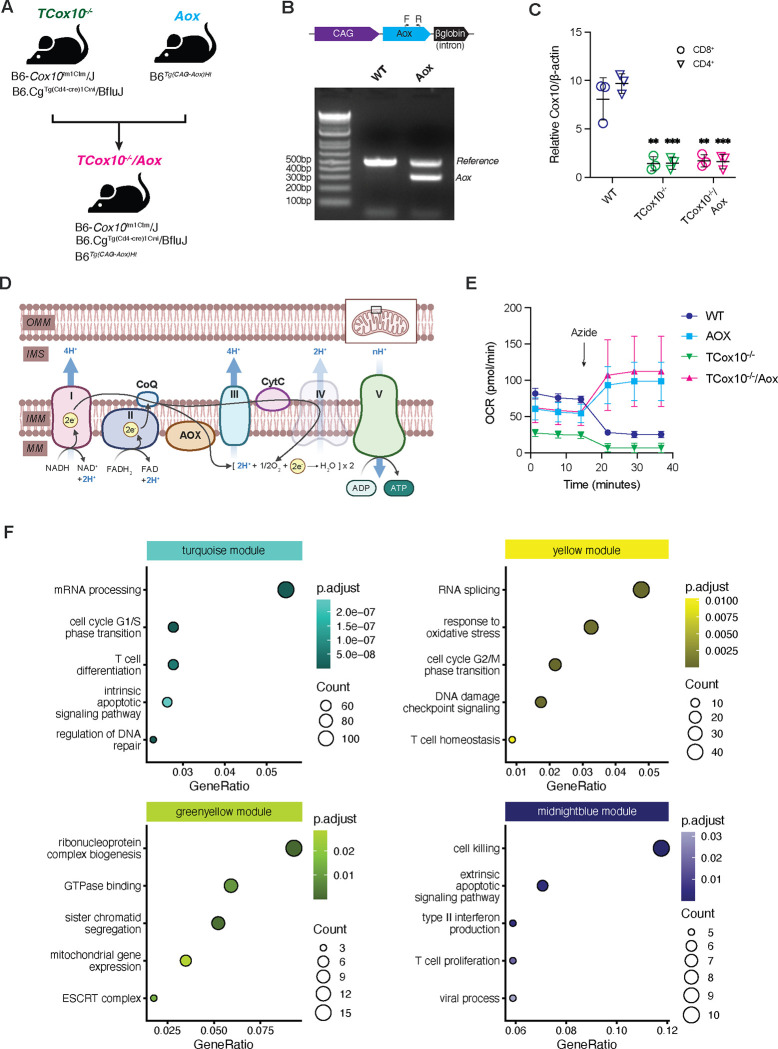
Alternative oxidase acts as a respiratory chain electron acceptor. (A) Schematic of mouse breeding scheme to generate TCox10^−/−^/Aox mice. In brief, mice with CD4-Cre driven knockout of *Cox10* (TCox10^−/−^ mice) were crossed with mice with systemic AOX expression to generate TCox10^−/−^/Aox mice. (B) Schematic of *Aox* transgene (top) and PCR confirmation of successful expression of *Aox*. (C) qPCR quantification of *Cox10* expression in both TCox10^−/−^ and TCox10^−/−^/AOX mice. (D) Schematic of mitochondrial respiratory chain with exogenous expression of alternative oxidase (AOX). In the absence of complex IV activity (in TCox10^−/−^ mice), AOX accepts electrons passed from complexes I and II to generate water and maintain proton motive force for ATP production. OMM; outer mitochondrial membrane, IMS; inner membrane space, IMM; inner mitochondrial membrane, MM; mitochondrial matrix. (E) Extracellular flux analysis of oxygen consumption rate (OCR) in T cells following treatment with 0.25mM sodium azide. (F) Overrepresentation analysis of four WGCNA modules. Selected enriched pathways are shown for each module. Point color reflects B-H adjusted p value, size reflects enriched genes in the set. Data are representative of one to three independent experiments and indicate mean and standard deviation. (C,E-F) n = 3–5 mice. * p < 0.05, ** p < 0.01, *** p < 0.001 by one-way ANOVA and post-hoc Dunnett test against WT. Specific *p* values (left to right) are as follows. (C) *CD8*^*+*^, F(2,6) = 22.6, p = 0.0016, Dunnett p = 0.0018, p = 0.0023; *CD4*^*+*^, F(2,6) = 99.59, p < 0.0001, Dunnett p < 0.0001, < 0.0001

COX10, a protoheme:heme-O-farnesyl transferase is indispensable for the biosynthesis of heme a, an elemental component of COX. Deficiency of COX10 results in marked impairment of COX and cellular respiration^9^; the molecular pathology of our previously published model in T cells (*TCox10*^−/−^). To restore MR specifically, we introduced a ubiquinol oxidase (AOX) from *Ciona intestinalis* into *TCox10*^−/−^ T cells. We engineered this novel mouse model by breeding *TCox10*^−/−^ mice with counterparts constitutively expressing the *Aox* gene to generate *TCox10*^−/−^*/Aox* mice (Fig. 1A), with PCR and qPCR analysis confirming the genotypes and expression of *Cox10* in the progeny (Fig. 1B and 1C). As AOX is an electron acceptor upstream of complex III (Fig. 1D), AOX-expressing cells can resist the effects of sodium azide, a toxin for COX, and continue respiration unaffected^7, 10, 11^. T cells derived from WT, *Aox*, *TCox10*^−/−^, and *TCox10*^−/−^*/Aox* mice were stimulated for 3 days with anti-CD3/CD28 and subjected to sodium azide. AOX-expressing cells maintained elevated oxygen consumption rates despite COX inhibition^10^, in contrast to the expected decline in WT and *TCox10*^−/−^ T cells (Fig. 1E).

To survey how AOX-mediated restoration of MR may impact cellular dynamics, we performed an RNAseq experiment using T cells from WT, *Aox*, *TCox10*^−/−^, and *TCox10*^−/−^*/Aox* mice. Each genotype showed significant changes in gene expression relative to WT, or in *TCox10*^−/−^*/Aox* against *TCox10*^−/−^ (Extended Data Fig. 1A and Extended Data Table 1). As these comparisons suggested considerable changes in gene expression across the four genotypes, we performed a weighted gene coexpression network analysis (WGCNA) to simultaneously compare them. Hierarchical clustering grouped the genes into 18 modules (Extended Data Fig. 1B). Examining the correlation of each module’s eigengene intercepts allowed us to identify which modules had gene expression perturbed in *TCox10*^−/−^ and normalized by the introduction of AOX (Extended Data Fig. 1C). We selected four modules (turquoise, yellow, greenyellow, and midnightblue) that followed this pattern and used a heatmap of the normalized gene expression from each category to confirm the differences in expression across groups (Extended Data Fig. 1D). Overrepresentation analysis (ORA) of each of the modules indicated the main functions of the modules in these gene sets (Extended Data Table 2), which included cell cycle phase transition, apoptotic signaling, and T cell function (Fig. 1F). To further elucidate the functional implications of AOX-mediated restoration in MR, we proceeded to validate these gene expression changes through targeted biochemical and functional assays.

### MR via AOX restores multiple aspects of mitochondrial function

**Fig 2.**
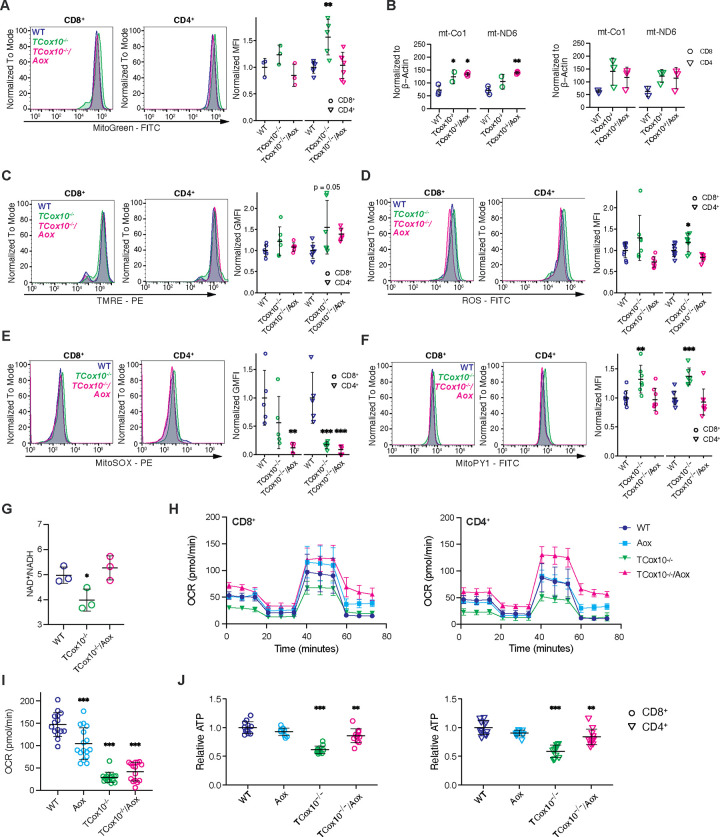
AOX restores mitochondrial function. (A) MitoGreen fluorescence in CD8^+^ and CD4^+^ T cells. Left, representative density plot; right, quantification of normalized mean fluorescence intensity (MFI). (B) qPCR quantification of mtDNA copy number. Left, CD8^+^, right, CD4^+^. (C) TMRE fluorescence in CD8^+^ and CD4^+^ T cells. Left, representative density plot; right, quantification of normalized geometric MFI. (D) ROS fluorescence in CD8^+^ and CD4^+^ T cells. Left, representative density plot; right, quantification of normalized MFI. (E) ROS fluorescence in CD8^+^ and CD4^+^ T cells. Left, representative density plot; right, quantification of normalized geometric MFI. (F) MitoPY1 fluorescence in CD8^+^ and CD4^+^ T cells. Left, representative density plot; right, quantification of normalized MFI. (G) NAD^+^/NADH ratio in T cells. (H) MitoStress Test measuring respiratory chain activity of CD8^+^ (left) and CD4^+^ (right) T cells. (I) Complex IV activity determined by oxygen consumption rate (OCR) following the addition of TMPD. (J) Relative ATP levels in CD8^+^ (left) and CD4^+^ (right) T cells. Data are representative of 1–3 experiments and indicate mean and standard deviation. (A-F) n = 2–9 mice. (G-J) n = 2–4 mice. * p < 0.05, ** p < 0.01, *** p < 0.001 by one-way ANOVA and post-hoc Dunnett test against WT. Specific *p* values (left to right) are as follows. (A) *CD4*^*+*^, F(2,15) = 9.249, p = 0.00242, Dunnett p = 0.0029, (B) *CD8*^*+*^, Co1, F(2,5) = 10.1, p = 0.018, Dunnett p = 0.048, 0.0142, Nd6, F(2,5) = 12.93, p = 0.011, Dunnett p = 0.007. (C) *CD4*^*+*^, F(2,15) = 3.12, p = 0.074, Dunnett p = 0.051. (D) *CD4*^*+*^, F(2,21) = 10.29, p = 7.7×10^−4^, Dunnett p = 0.045. (E) *CD8*^*+*^, F(2,12) = 6.41, p = 0.013, Dunnett p = 0.007; *CD4*^*+*^, F(2,12) = 18.23, p = 2.3×10^−4^, Dunnett p = 6.3×10^−4^, 2.7×10^−4^. (F) *CD8*^*+*^, F(2,21) = 8.17, p = 0.0024, Dunnett p = 0.0047; *CD4*^*+*^, F(2,21) = 14.69, p = 1.02×10^−4^, Dunnett p = 5.4×10^−4^. (G) F(2,6) = 7.735, p = 0.022, Dunnett p = 0.048. (I) F(3,58) = 72.85, p < 0.0001, Dunnett p < 0.0001, p < 0.0001, p < 0.0001. (J) *CD8,* F(3,44) = 39.45, p = 1.55×10^−12^, Dunnett p = 6.4×10^−13^, 1.5×10^−3^; *CD4,* F(3,44) = 31.61, p = 4.71×10^−11^, Dunnett p = 2.9×10^−12^, 2.1×10^−3^.

After confirming the activity of AOX in T cells, we subsequently concentrated on mitochondrial function, given that this cellular organelle is the primary site of its activity. Transcriptional profiling of *TCox10*^−/−^*/Aox* mice revealed restoration of OXPHOS gene set enrichment, particularly gene sets associated with complex I (Extended Data Fig. 2A). Additionally, there were substantial enhancements in transcriptional signatures related to mtDNA metabolism and translation processes, as well as carbohydrate and nucleotide metabolism (Extended Data Fig. 2A and Extended Data Table 3). In *TCox10*^−/−^*/Aox* T cells, mitochondrial number (MitoGreen, Fig. 2A) reverted to WT levels. However, mtDNA content by qPCR (Fig. 2B) remained increased, suggesting that cells were still compensating for COX deficiency. Indeed, COX has been demonstrated to modulate mitochondrial genomic homeostasis,^5^ a process which may be independent of MR, as indicated by our results.

Based on its position in the ETC, we hypothesized that COX-mediated MR functions as a regulatory mechanism to mitigate the build-up of electron pressure within the ETC by acting as a safety valve. This effectively reduces the formation of reactive oxygen species (ROS). *TCox10*^−/−^ T cells tend to have higher and more variable membrane potential (TMRE, Fig. 2C) suggesting an increase in electron build-up. *TCox10*^−/−^*/Aox* T cells show a normalization of mitochondrial membrane potential, similar to WT levels. This relief of electron build-up is further highlighted in cellular ROS. In *TCox10*^−/−^ T cells, we observed elevated total cellular ROS (Fig. 2D), in the presence of diminished superoxide (Fig. 2E) and elevated hydrogen peroxide (Fig. 2F). These findings not only indicated oxidative stress, but also augmented superoxide dismutase activity (i.e., superoxide → hydrogen peroxide). AOX, which bypasses complex III, a major source of ROS in T cells^3^, reduced ROS production, and hydrogen peroxide, indicating a restoration in redox balance. This was further supported by the AOX-mediated normalization of NAD^+^/NADH ratios, further signifying a restoration of cellular redox, and demonstrating the efficacy of AOX in alleviating electron pressure in the ETC (Fig. 2G).

We next turned to evaluating cellular respiration by extracellular flux analysis. OCR was markedly improved with AOX in CD4^+^ and CD8^+^ T cells with *TCox10*^−/−^*/Aox* cells exceeding that of WT (Fig. 2H and Extended Data Fig. 2B). To demonstrate that the observed increase in OCR did not relate to COX activity, we used a targeted substrate (N′-tetramethyl-para-phenylene-diamine (TMPD)). COX-mediated respiration remained low in *TCox10*^−/−^*/Aox* T cells, suggesting that AOX does not enhance COX activity but instead functions independently (Fig. 2I). AOX addition also resulted in the augmentation of total cellular ATP production, although not to WT levels (Fig. 2J). Therefore, reestablishing by AOX cellular respiration reduces oxidative stress, and improves ATP production and cellular redox balance, restoring mitochondrial homeostasis, ultimately revealing insights into the role of COX-dependent respiration in the T cell processes.

### MR offloads upstream carriers of chemical energy during metabolic reprogramming

Based on our finding that MR reduces electron buildup and restores the cellular redox state (i.e., ROS and NAD^+^/NADH), we next asked whether this function also serves as a safety valve for upstream metabolic processes. The TCA cycle and glycolysis are integral to T cell metabolic reprogramming^2^ and are dependent on the redox state of the cell. To better understand the role of MR in supporting upstream metabolic function, we employed stable isotope tracing experiments during metabolic reprogramming in 24 hour activated T cells, as above.

**Figure 3.**
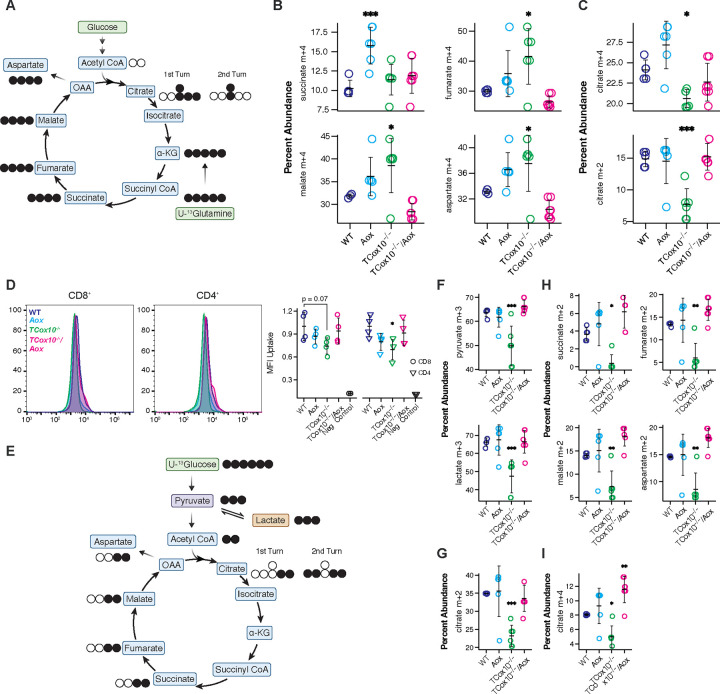
AOX normalizes aberrant TCA substrate flux. (A) Schematic of stable isotope labeling of TCA metabolites from U-^13^glutamine. Black circles indicate labeled carbons, white circles indicate unlabeled carbons. (B) Quantification of succinate, fumarate, malate, and aspartate (m+4) abundance deriving from glutamine across genotypes. (C) Quantification of m+4 citrate (first TCA turn) and m+2 citrate (second TCA turn) abundance deriving from glutamine across genotypes. (D) 2-NDBG uptake analysis in CD8+ and CD4+ T cells. Left, representative density plots; right, quantification of mean fluorescence intensity (MFI) across genotypes. (E) Schematic of stable isotope labeling of TCA metabolites from U-^13^glucose. Black circles indicate labeled carbons, white circles indicate unlabeled carbons. (F) Quantification of pyruvate and lactate (m+3) abundance deriving from glucose. (G) Quantification of citrate (m+2) abundance deriving from glucose. (H) Quantification of succinate, fumarate, malate, and aspartate (m+2) abundance deriving from glucose. (I) Quantification of citrate (m+4) abundance deriving from glucose. Data are representative of 1–2 experiments and indicate mean and standard deviation. (B-C) n = 5–7 mice, (D) n = 4 mice, (F-I) n = 5–7 mice. * p < 0.05, ** p < 0.01, *** p < 0.001 by one-way ANOVA and post-hoc Dunnett test against WT. Specific *p* values (left to right) are as follows. (B) *succinate*, F(3,20) = 7.96, p = 0.0011, Dunnett p = 0.0007; *fumarate*, F(3,20) = 7.417, p = 0.0016, Dunnett p = 0.0144; *malate,* F(3,20) = 9.076, p = 0.0005, Dunnett p = 0.022; *aspartate,* F(3,20) = 9.753, p = 0.0004, Dunnett p = 0.033, (C) *citrate m+4*, F(3,20) = 10.77, p = 0.0002, Dunnett p = 0.0277; *citrate m+2,* F(3,20) = 12.29, p < 0.0001, Dunnett p = 0.0004. (D) *CD8*^*+*^, F(3,12) = 2.36, p = 0.123, Dunnett p = 0.0661; *CD4*^*+*^, F(3,12) = 3.702, p = 0.0428, Dunnett p = 0.0233. (F) *pyruvate*, F(3,20) = 13.32, p < 0.0001, Dunnett p = 0.0005; *lactate* F(3,20) = 11.19, p = 0.0002, Dunnett p = 0.0008, (G) F(3,20) = 10.95, p = 0.0002, Dunnett p = 0.0006, (H) *succinate*, F(3,20) = 10.11, p = 0.0003, Dunnett p = 0.0194; *fumarate,* F(3,20) = 12.86, p < 0.0001, Dunnett p = 0.0029; *malate*, F(3,20) = 13.86, p < 0.0001, Dunnett p = 0.0045; *aspartate,* F(3,20) = 14.86, p < 0.0001, Dunnett p = 0.003, (I) F(3,20) = 15.6, p < 0.0001, Dunnett p = 0.0223.

Glutamine is an anaplerotic amino acid in the TCA cycle, generating reducing equivalents that can drive OXPHOS^12, 13^. Previously, we demonstrated that activated *TCox10*^−/−^ T cells develop glutamine addiction with increased incorporation of this amino acid into the TCA cycle^4^. In *TCox10*^−/−^*/Aox* T cells, we observed that the incorporation of ^13^C-glutamine carbons into downstream metabolites (Fig. 3A), including fumarate (M + 4), malate (M + 4), and aspartate (M + 4), decreased, returning to WT levels (Fig. 3B) and indicating the resolution of glutamine addiction. Citrate (M + 4 and M + 2) concentrations were also improved, signaling a reactivation of cycling dynamics of the TCA (Fig. 3C).

*TCox10*^−/−^ T cells also demonstrated depressed glycolysis following activation^4^, driven in part by negative enrichment and downregulation of glycolytic pathway genes (Extended Data Fig. 2A). To study glycolysis, we first examined glucose transport, a process that is upregulated in T cells during metabolic reprogramming^14^. While glucose transport via 2-NDBG uptake was suppressed in activated *TCox10*^−/−^ T cells, this process was reestablished in *TCox10*^−/−^*/Aox* CD4^+^ and CD8^+^ T cells (Fig. 3D). With enhanced glucose transport in *TCox10*^−/−^*/Aox* T cells, we next focused on the fate of glucose carbons using stable isotope tracing with ^13^C-glucose (Fig. 3E). Glucose free media was supplemented with ^13^C-glucose and T cells were stimulated for 24 hours as above. *TCox10*^−/−^*/Aox* T cells displayed a normalized uptake of ^13^C into pyruvate (M + 3) and lactate (M + 3), signaling a restoration of glycolytic activity (Fig. 3F). Interestingly, glycolytic gene expression is not restored in *TCox10*^−/−^*/Aox* T cells (Extended Data Fig. 2A), suggesting that this enhancement occurs through post-transcriptional mechanisms. We next asked whether the conversion of glucose into the TCA cycle via pyruvate also returned. Indeed, we observed elevated ^13^C incorporation into citrate (M + 2) signifying enhanced influx of glucose-derived carbons into the TCA cycle (Fig. 3G). The enrichment of downstream TCA cycle intermediates—succinate, fumarate, malate, and aspartate (all M + 2)—further confirmed this restoration (Fig. 3H). An enhancement in the isotopic enrichment of citrate (M + 4) also highlighted the resumption of cycling of the TCA (Fig. 3I). Thus, by enabling the flow of electrons within the ETC, MR reduces the upstream electron pressure from glutaminolysis and glycolysis concomitantly maintaining TCA cycle function in T cells. Our results also suggest that a significant portion of glucose is eventually oxidized in the mitochondria during T cell activation.

### MR abrogates apoptosis

**Figure 4.**
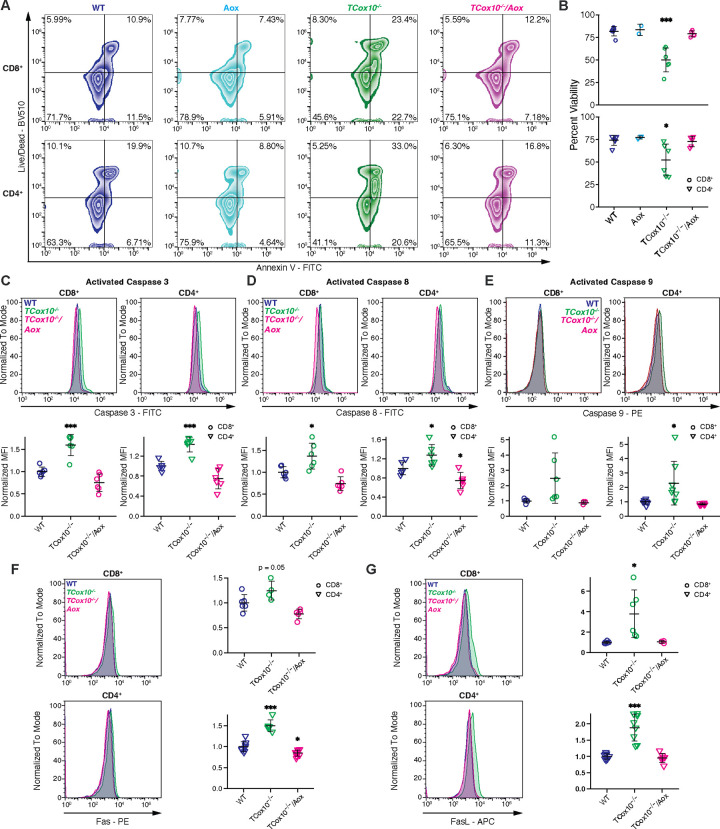
AOX abrogates TCox10−/− proliferation-induced apoptosis. (A) Representative contour plot of viability analysis in CD8^+^ and CD4^+^ T cells using Live/Dead and Annexin V stains. (B) Quantification of viability analysis. (C) Caspase 3 activity in CD8^+^ and CD4^+^ T cells. Top, representative density plot; bottom, quantification of normalized mean fluorescence intensity (MFI). (D) Caspase 8 activity in CD8^+^ and CD4^+^ T cells. Top, representative density plot; bottom, quantification of normalized MFI. (E) Caspase 9 activity in CD8^+^ and CD4^+^ T cells. Top, representative density plot; bottom, quantification of normalized MFI. (F) Fas expression in CD8^+^ and CD4^+^ T cells. Left, representative density plot; right, quantification of normalized MFI. (G) FasL expression in CD8^+^ and CD4^+^ T cells. Left, representative density plot; right, quantification of normalized MFI. Data are representative of two to three independent experiments and indicate mean and standard deviation. (A-D) n = 2–6 mice, (F-G) n = 3–9 mice. * p < 0.05, ** p < 0.01, *** p < 0.001 by one-way ANOVA and post-hoc Dunnett test against WT. Specific *p* values (left to right) are as follows. (B) *CD8*^*+*^, F(3,15) = 18.15, p = 2.97×10^−5^, Dunnett p = 3.8×10^−5^; *CD4*^*+*^, F(3,15) = 5.26, p = 0.011, Dunnett p = 0.0114, (C) *CD8*^*+*^, F(2,15) = 32.25, p = 3.7×10^−6^, Dunnett p = 0.00011; *CD4*^*+*^, F(2,15) = 30.62, p = 5.05×10^−6^, Dunnett p = 0.0004, 0.0212, (D) *CD8*^*+*^, F(2,15) = 14.1, p = 0.00036, Dunnett p = 0.014; *CD4*^*+*^, F(2,15) = 12.98, p = 0.000534, Dunnett p = 0.034, 0.0484, (E) *CD4*^*+*^, F(2,21) = 6.466, p = 0.00649, Dunnett p = 0.0123, (F) *CD8*^*+*^, F(2,12) = 10.48, p = 0.00233, Dunnett p = 0.0526; *CD4*^*+*^, F(2,21) = 66.97, p = 7.7×10^−10^, Dunnett p = 4.2×10^−8^, p = 0.0149, (G) *CD8*^*+*^, F(2,12) = 6.123, p = 0.0147, Dunnett p = 0.014; *CD4*^*+*^, F(2,21) = 33.1, p = 3.22×10^−7^.

In our previous study, we demonstrated that COX mediates apoptosis in activated T cells during the phase of proliferation^4^. We next asked whether the restoration of MR could restore cellular viability. Building on our previous findings, alterations in apoptosis were disentangled by mapping *TCox10*^−/−^*/Aox* versus *TCox10*^−/−^ log_2_ fold changes (L2FCs) from our RNAseq onto the KEGG apoptosis pathway (mmu04210) (Extended Data Fig. 3A). While multiple pro-apoptotic and pro-survival genes were upregulated in *TCox10*^−/−^ T Cells, interestingly, elevated expression of extrinsic activators *Fas*, *FasL*, *Perf1*, and *Tradd* was reversed by AOX. Apoptosis was measured in T cells by live/dead dye and Annexin V staining (Fig. 4A), revealing a reduction in apoptotic *TCox10*^−/−^*/Aox* CD4^+^ and CD8^+^ T cells by approximately 50% (Fig. 4B). To investigate potential mechanisms underlying the reduction in apoptosis, we assessed caspase 3 activation, a common pathway for both intrinsic and extrinsic apoptosis. Caspase 3 activation was abnormally elevated in *TCox10*^−/−^ T cells and decreased in *TCox10*^−/−^*/Aox* T cells (Fig. 4C). We also observed similar trends in specific apoptotic pathways, showing amelioration of both caspase 8 (i.e., extrinsic pathway, Fig. 4D) and caspase 9 (i.e., intrinsic pathway, Fig. 4E) activation in *TCox10*^−/−^*/Aox* T cells. Since we observed more consistent increased expression of Caspase 8 and given our RNAseq results (Extended Data Fig. 3A and Extended Data Table 1), we further examined activation of the extrinsic pathway by quantifying Fas and FasL (Figs. 4F and 4G). Expression of both proteins were increased in *TCox10*^−/−^ T cells and reduced with AOX expression. To probe the activity of this pathway *in vitro*, we used anti-FasL antibodies on WT and *TCox10*^−/−^ CD8^+^ and CD4^+^ T cells. This intervention significantly increased the viability of *TCox10*^−/−^ T cells (Extended Data Fig. 3B) indicating a major role for this pathway in COX mediated apoptosis. However, blocking FasL did not improve proliferation as reflected by the retention of Cell Trace Violet (CTV, Extended Data Fig. 3C) underscoring the importance of maintaining MR. Therefore, these data support that COX-mediated apoptosis involves the external pathway and is secondary to MR dysfunction in T cells.

### MR sustains T cell function in vitro

**Fig 5.**
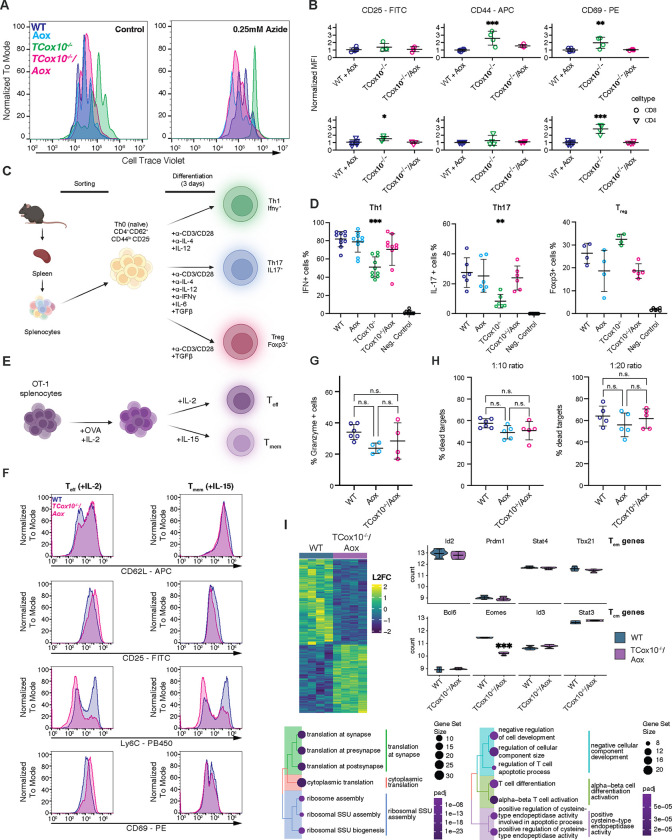
AOX restores TCox10−/− function in vitro. (A) Proliferation analysis (Cell Trace Violet) of T cells without and following treatment with 0.25mM sodium azide. (B) Surface expression of T cell activation markers, quantified by mean fluorescence intensity (MFI). (C) Schematic of T cell subtype isolation and differentiation. In brief, splenocytes are isolated from mouse spleen, sorted for naïve T cells (Th0) and activated with subtype-specific factors to differentiate into Th1, Th17, or Tregs. (D) Percentage of cells differentiated into Th1, Th17, or Tregs following activation. (E) Schematic of OT-1 splenocyte differentiation into Teff and Tmem subtypes. (F) Surface expression of T_eff_ and T_mem_ activation markers between IL-2 or IL-15 differentiated WT and TCox10^−/−^/Aox T cells. (G) Percent granzyme positive T cells across genotype. (H) Percent dead targets across genotype. (I) Differential expression between IL-15-differentiated WT and TCox10^−/−^/Aox T_mem_ cells. Top left, heatmap of significant differentially expressed genes. Top right, normalized expression of T_em_ and T_cm_ genes. Bottom left, ORA of significantly upregulated genes in TCox10^−/−^/Aox Tmem cells. Bottom right, ORA of significantly downregulated genes in TCox10^−/−^/Aox T_mem_ cells. Data are representative of at least two independent experiments (with the exception of RNAseq; one experiment) and indicate mean and standard deviation. (A-B), n = 3–8 mice, (D), n = 4–10 mice, (G-I), n = 4–5 mice. * p < 0.05, ** p < 0.01, *** p < 0.001 by one-way ANOVA and post-hoc Dunnett test against WT unless otherwise specified below. Negative controls are not included in statistical calculations. Specific *p* values (left to right) are as follows. (B), *CD8,* F(2,13) = 15.4, p = 0.000372, Dunnett p = 0.00018, F(2,13) = 10.08, p = 0.00228, Dunnett p = 0.0017; *CD4*, F(2,13) = 4.583, p = 0.0312, Dunnett p = 0.0255, F(2,13) = 41.73, p = 2.2×10^−6^, Dunnett p = 1.8×10^−6^, (D) F(3,36) = 12.04, p < 0.0001, Dunnett p = < 0.0001, F(3,20) = 6.04, p = 0.002, Dunnett p = 0.003 (I) Wald test t stat= −16.37, B-H adjusted p value = 4.31×10^−56^.

Significant deficiencies in T cell differentiation and function result from COX deficiency^4^. With a re-establishment of metabolic reprogramming and a suppression of apoptosis by AOX, we next evaluated core functions in *TCox10*^−/−^*/Aox* T cells in vitro. Following metabolic reprogramming, T cells engage in robust proliferation. To examine the impact of MR on proliferation, we loaded T cells with CTV and activated for 3 days as above. *TCox10*^−/−^*/Aox* T cell proliferation matched WT, even when challenged with sodium azide (Fig. 5A), indicating that the AOX-mediated MR could support proliferation. Flow cytometry analysis revealed that activated *TCox10*^−/−^ T cells displayed cell surface markers indicative of a heightened activation state (CD44, CD69, Fig. 5B) while *TCox10*^−/−^*/Aox* cells more closely aligned to WT.

To evaluate the role of MR in T cell differentiation, we produced helper T cells (Th) in *TCox10*^−/−^*/Aox* cells in vitro, following a standardized protocol (Fig. 5C). We found that *TCox10*^−/−^*/Aox* T cells acquired differentiation capacities, particularly in Th1 and Th17 subsets, approximating the patterns observed in WT cells (Fig. 5D). Regulatory T cells (Foxp3^+^, T_reg_), were decreased in AOX expressing cells, signifying a more complex relationship between mitochondrial function and their differentiation.

We next evaluated T cell effector (T_eff_) and memory (T_mem_) differentiation in vitro, essential processes for sustained immune protection (Fig. 5E). In *TCox10*^−/−^ T cells, memory and effector cell differentiation were previously unachievable due to overwhelming apoptosis and death. The re-introduction of MR facilitated the generation of both T_eff_ and T_mem_. These cells exhibited improvements in the expression of phenotypic markers of differentiation for their respective cell types, albeit incompletely (Fig. 5F). To assess the functionality of T_eff_ cells, we stained for the expression of granzyme, an essential molecule for cytolytic activity. Granzyme levels in *TCox10*^−/−^*/Aox* matched those in WT and *Aox* controls, and their killing activity was similarly robust, confirming that the cytotoxic capabilities were fully present (Figs. 5G and 5H). Given the lack of a robust in vitro assay for memory function, we performed RNAseq analysis on *TCox10*^−/−^*/Aox* differentiated memory T cells (Fig. 5I and Extended Data Table 4). Despite significant differences in their transcriptional profiles, the expression of core genes involved in effector memory (T_em_) and central memory (T_cm_) differentiation was largely similar between *TCox10*^−/−^*/Aox* and WT cells, with the notable exception of Eomes. This indicates that while *TCox10*^−/−^*/Aox* memory T cells exhibit broad transcriptional changes, essential pathways for memory differentiation mostly remain intact. All things considered, our findings in *TCox10*^−/−^*/Aox* T cells underscore that maintaining MR, the core function of COX, is essential for T cell differentiation. Following these encouraging results in T_eff_ and T_mem_, it became imperative to further assess the functional capabilities of these cells in a physiologically relevant setting (i.e., in vivo).

### MR is critical for T cells in vivo

**Fig 6.**
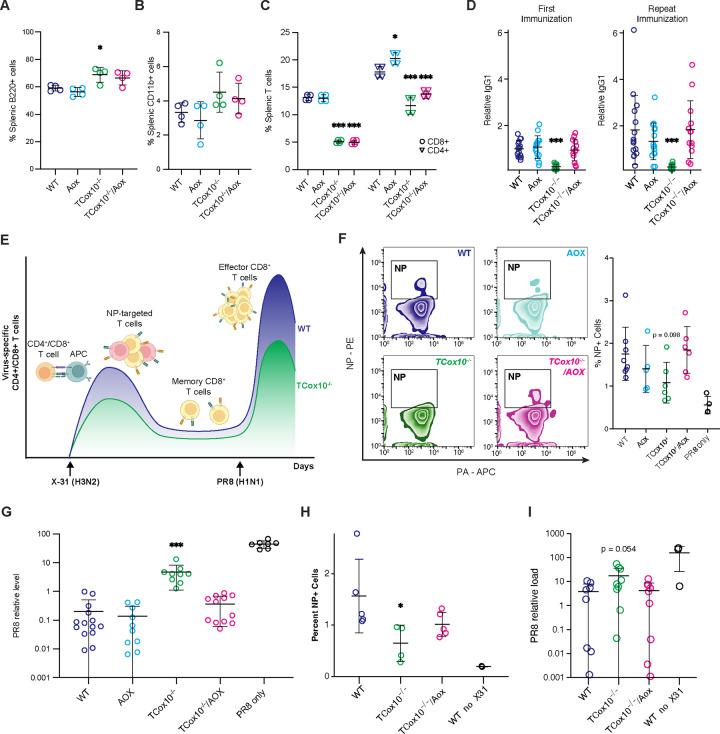
Aox restores T cell function in vivo. (A) Percentage of B220^+^ B cells isolated from spleen. (B) Percentage of CD11b^+^ cells isolated from spleen. (C) Percentage of CD8^+^ or CD4^+^ T cells isolated from spleen. (D) Relative T cell-dependent antigens (IgG1) detected after first immunization (left) and reimmunization (right). (E) Schematic of deficient development of effector CD8^+^ T cells (T_eff_) in *TCox10*^−/−^ mice following viral antigen rechallenge. Mice are initially exposed to X-31, a mouse-adapted non-lethal H3N2 influenza virus, which stimulates the development of T cells targeted to X-31 peptides, including nucleoprotein (NP). Antigen-specific memory CD8^+^ T cells (T_mem_) persist following the initial infection. Mice are then exposed to PR8, a mouse-adapted lethal H1N1 influenza virus, which shares the NP antigen with X-31. In wildtype mice, this stimulates the generation of abundant NP-targeted T_eff_ cells, but in *TCox10*^−/−^mice this response is diminished. (F) Left, contour plot of NP and PA positivity in CD8^+^ T cells isolated from X31/PR8-exposed mice. Right, quantification of percent NP-positive CD8^+^ T cells. (G) PR8 relative viral load in X31/PR8-exposed mice. (H-I) Bone marrow transfer experiment wherein bone marrow from CD45.2 mice of indicated genotypes replaces bone marrow from CD45.1 WT mice. (H) PR8 relative viral load following X31/PR8-exposure. (I) Percent NP-positive CD8^+^ T cells following X31/PR8-exposure. Data are representative of one to four independent experiments and indicate mean and standard deviation. (A-C) n = 4 mice, (D) n = 7–13 mice, (F) n = 5–8 mice, (G) n = 9–14 mice, (H-I) 4–9 mice. * p < 0.05, ** p < 0.01, *** p < 0.001 by one-way ANOVA and post-hoc Dunnett test against WT unless otherwise specified below. Controls are not included in statistical calculations. Specific *p* values (left to right) are as follows. (A), F(3,12) = 8.318; p = 0.0029, Dunnett p = 0.0160, (C) CD8 F(3,12) = 382.5, p < 0.0001, Dunnett p < 0.0001, p < 0.0001; CD4 (F3,12) = 46.22, p < 0.0001, Dunnett p = 0.0263, p < 0.0001, p = 0.0009, (D) *First immunization,* F(3,50) = 11.65, p = 6.61×10^−6^, Dunnett p = 4.4×10^−5^; *Repeat immunization,* F(3,50) = 5.81, p = 0.002, Dunnett p = 0.0015. (F) F(3,21) = 2.414, p = 0.0952. (G) F(3,41) = 18.75, p = 8.21×10^−8^, Dunnett p = 1.3×10^−7^, (H) F(2,23) = 3.628, p = 0.0427, (I) F(2,11) = 4.036, p = 0.0485, Dunnett p = 0.032.

T cells orchestrate and execute the immune response through cytokine production or direct cellular interactions, serving as both regulators and effectors. A core function of T cells is maintaining immune memory, ensuring a rapid and effective response to previously encountered antigens. To evaluate how MR may support these diverse abilities, we studied *TCox10*^−/−^*/Aox* T cell development and function in vivo. As a secondary lymphoid organ, the spleen serves as a microenvironment for immune interactions, providing a specialized niche where immune cells such as T cells, B cells, and macrophages coordinate responses. In the spleens of both *TCox10*^−/−^ and *TCox10*^−/−^*/Aox* mice, we observed a tendency for elevated B cells and macrophages, signifying imbalances between splenic resident cells (Figs. 6A and 6B). Despite the AOX, decreased quantities of both CD4^+^ and CD8^+^ T cells persisted in the spleens of *TCox10*^−/−^*/Aox* mice (Fig. 6C). This reduction signals that AOX, while improving some aspects of T cell function, cannot fully restore T cell populations to normal levels. With these numeric perturbations in splenic populations, we next measured T-dependent B cell responses through immunization with 2,4,6-Trinitrophenyl-Chicken Gamma Globulin (TNP-CGG). The results were promising: unlike *TCox10*^−/−^, *TCox10*^−/−^*/Aox* mice mounted effective primary (2 weeks) and secondary (5 weeks) B cell responses, illustrating that MR enhances the supportive role of T cells (Fig. 6D).

Mitochondria are integral to the development of T cell memory by providing the necessary energy and signaling pathways that support their long-term survival and rapid response capabilities^15^. To assess the requirement of MR for development of memory T cells, we conducted an in vivo challenge with influenza virus (Fig. 6E). Mice were first immunized with influenza A/X31 (X31, H3N2) followed with influenza A/PR/8 (PR8, H1N1) challenge at 5 weeks. Both immunization and challenge were conducted using inhalation. Our experimental design, based on a switch in viral isotypes (X-31→PR8), eliminates the memory humoral responses, allowing us to focus on T cells. To demonstrate the generation of influenza-specific memory, we stained T cells with tetramers against two T cell antigenic determinants, the nucleoprotein (NP_366–374_, H2Db) and the acid polymerase (PA_224–233_, H2Db)^16, 17^. The primary CD8^+^ T cell response to both strains is dominated by naïve T-cell recognition of both determinants. However, the NP_366–374_ response dominates the secondary response in X-31→PR8 isotype switch challenge. *TCox10*^−/−^ mice showed a limited ability to generate memory cells, while *TCox10*^−/−^*/Aox* mice successfully generated memory T cells at levels comparable to WT (Fig. 6F). The most direct evidence of functional recovery came from viral load assessments, where *TCox10*^−/−^*/Aox* mice exhibited viral loads not significantly different from WT, significantly lower than those seen in *TCox10*^−/−^ mice (Fig. 6G). This dramatic reduction in viral load highlights the restored antiviral efficacy of T cells in AOX-expressing mice. Lastly, to ascertain whether the improvements were cell-autonomous, we conducted adoptive transfer experiments using bone marrow from WT, *Aox*, *TCox10*^−/−^, and *TCox10*^−/−^*/Aox* mice. We found that mice reconstituted with *TCox10*^−/−^*/Aox* bone marrow could produce added viral specific T cells (Fig. 6H) and reduce viral load to levels similar to WT (Fig. 6I), demonstrating that the benefits of AOX were indeed cell-autonomous.

## DISCUSSION

In this study, we introduced an AOX from *Ciona intestinalis* into *TCox10*^−/−^ T cells to investigate the role of MR in T cell function. Our findings reveal that MR serves as a safety valve to manage electron pressure from metabolic reprogramming during T cell activation. Restoring MR reinstates the cellular redox state and eliminates the secondary effects of COX dysfunction: apoptosis and metabolic perturbations. By providing this metabolic adaptability, MR ensures robust T cell activation and function, emphasizing the importance of maintaining COX integrity.

Ubiquinol, the reduced form of ubiquinone (coenzyme Q), is vital in mammalian cells, facilitating electron transfer from complexes I and II to complex III in the ETC, essential for ATP production and maintaining mitochondrial membrane potential. Moreover, it is integral to redox regulation, balancing oxidized and reduced states central to cellular signaling, metabolic processes, and stress-induced apoptosis^18, 19^. The job of ubiquinol in cell growth is also notable, as its oxidation supports the TCA cycle and de novo pyrimidine synthesis, essential for cell proliferation^8^. COX interacts with the ubiquinol pool by ensuring the final transfer of electrons to oxygen in the ETC. This step is indispensable for the continuous oxidation of ubiquinol, thereby maintaining the flow of electrons through the ETC and supporting mitochondrial functions.

In *TCox10*^−/−^*/Aox* T cells, AOX becomes a crucial component in the mitochondrial ETC, providing an azide-resistant pathway that bypasses complexes III and IV. By oxidizing ubiquinol to ubiquinone and reducing oxygen to water, AOX effectively prevents the over-reduction of the ubiquinone pool and mitigates ROS formation, thus protecting cells from oxidative stress^20^. The activity of AOX is tightly regulated by the redox state of the ubiquinone pool, becoming significantly active when the pool is more than 35–40% reduced^21, 22^. This regulation allows AOX to function as an “energy overflow” mechanism, facilitating the continuous shunting of excess electrons when the cytochrome pathway is saturated or inhibited, as in *TCox10*^−/−^ T cells. Similarly, COX plays a vital role in maintaining the redox state of the ubiquinol pool and preventing the over-reduction that leads to ROS formation. By ensuring that the ubiquinone pool remains properly balanced, COX prevents the accumulation of excess electrons, which can result in ROS and cellular damage. COX acts as an overflow mechanism, much like AOX, ensuring efficient electron flow within the electron transport chain and protecting cells from oxidative stress.

T cells undergo metabolic reprogramming to meet the demands of activation and function, dynamically shifting between glycolysis, the TCA cycle, FAO and OXPHOS. Notably, the Warburg effect, a shift to glycolysis even under aerobic conditions upon activation, ensures rapid energy production. However, mitochondrial dysfunction, as seen in COX-deficient T cells, can severely impair these metabolic pathways, affecting T cell responses and leading to immunodeficiency due to compromised metabolic functionality^4^. By providing this offloading pathway for reducing equivalents, MR by AOX ensures more efficient operation of both glycolysis and the TCA cycle. Consequently, AOX enhances cellular energy production and reduces oxidative stress, playing a pivotal role in restoring metabolic functionality, cellular energy balance and stress adaptation^23, 24^.

COX has multiple functions, however the transfer of electrons from cytochrome c to molecular oxygen sustains the ETC^25^. This process facilitates the translocation of protons from the mitochondrial matrix to the intermembrane space, creating an electrochemical gradient necessary for ATP synthesis^26, 27^. The inability to perform this core function leads to release of cytochrome c from mitochondria into the cytoplasm, triggering apoptotic pathways^28^. In *TCox10*^−/−^ T cells, overwhelming apoptosis leads to a T cell mediated immunodeficiency^4^. While apoptosis is a significant outcome of COX deficiency, by introducing the AOX, we show that its primary role is in MR. All other pathologies of COX deficiency stem from the loss of MR. Besides mediating apoptosis and acting as a metabolic safety valve as discussed above, MR also modulates mtDNA and nDNA transcription and maintenance of mtDNA^5^ as supported by our RNAseq and mtDNA studies. The impact on pathways related to the cell cycle, proliferation, and lymphocyte differentiation, migration, and activation in *TCox10*^−/−^*/Aox* T cells demonstrates the broad effects of MR. It should be noted however, that COX deficiency, and resultant impairment of MR, is not just limited to genetic models. Inhibition of COX is mediated through diverse mechanisms which can occur in immune niches including chemical inhibition by drug complexes, ionic competition, and physiological regulatory molecules like nitric oxide and ATP^29, 30, 31, 32^, making our findings broadly applicable.

By demonstrating the compensatory role of AOX, we establish the primary function of COX in T cells as MR. The prevention of ROS overproduction, metabolic perturbations, apoptosis, and T cell dysfunction by AOX emphasizes that these are secondary pathologies of COX deficiency. Our results underscore the importance of maintaining COX integrity for overall cellular health, highlighting the pivotal role of MR in energy production, transcriptional regulation, and cellular proliferation and differentiation.

## METHODS

### Mouse Lines

C57Bl/6J (WT, B6) (strain #000664), Tg(Cd4-cre)1Cwi/BfluJ (Cd4-cre) (strain #017336), and C57BL/6-Tg(TcraTcrb)1100Mjb/J (OT-1) (strain #003831) mice were purchased from Jax. B6.129X1-Cox10^tm1Ctm^/J (Cox10^fl/fl^) were a kind gift from C. Morales and F.Diaz (University of Miami). *Rosa26-*targeted *Ciona intestinalis* alternative oxidase expressing (Aox) mice were a generous gift from Drs. Marten Szibor and Howard T. Jacobs^33^. All procedures involving animals were in compliance with the Animal Care and Use Committee of the National Human Genome Research Center under an established protocol (G-11–3).

### Cell Culture

Bulk T cell preparations were isolated from spleen with Pan-T cell Separation kit (Miltenyi Biotec) and stimulated *ex vivo* for 24 or 72 hours with anti-CD3/CD28 (BioXCell) before collection. For effector and memory T cell preparation, splenocytes from mice crossed with OT1 transgenic mice were isolated, treated *ex vivo* for three days with OVA peptide, followed by treatment for three days with IL-2 or IL-15 before collection as indicated in the text. Pan T cells, CD8^+^ T or CD4^+^ cells were enriched using isolation kits (Miltenyi Biotec). Purity of T cells was > 95% in all cases. T cells were stimulated with plate-bound anti-CD3 (5 μg/ml) and anti-CD28 (0.5 μg/ml). Cells were treated sodium azide with varying concentrations as described in the results.

### Stable Isotopes

Mouse T cells were stimulated for 24 hr with plate-bound anti-CD3/CD28. Labeling experiments were performed essentially as described previously^34, 35^. All labeling experiments were performed with 1 million cells/mL cultured in RPMI containing 11 mM glucose and 2 mM glutamine, with one nutrient or the other replaced by a uniformly ^13^C-labeled analog (i.e., [U−^13^C] glucose or [U−^13^C] glutamine; Cambridge Isotope Laboratories). Cells were rinsed in phosphate-buffered saline, then replenished with labeling medium at time 0. Culture proceeded for 24 hr, then the cells were briefly rinsed in cold saline, pelleted, and lysed in cold 50% methanol. The lysates were subjected to at least three freeze-thaw cycles, then centrifuged to remove debris. The supernatants were evaporated to dryness, methoximated and derivatized by tert-butyl dimethylsilylation. One μL of the derivatized material was injected onto an Agilent 6970 gas chromatograph equipped with a fused silica capillary GC column (30 m length, 0.25 mm diameter) and networked to either an Agilent 5973 or a 5975 Mass Selective Detector. Retention times of all metabolites of interest were validated using pure standards. The measured distribution of mass isotopomers was corrected for natural abundance of ^13^C.^36^

### RNAseq

T cells (10^6^ cells) were lysed in TRIzol (Thermo Fisher). Total RNA was isolated and purified with RNeasy kit (Qiagen) according to manufacturer’s protocol. RNAseq was performed by an outside commercial laboratory (Novogene, Sacramento, CA). Messenger RNA was purified using poly-T oligo-attached magnetic beads. First strand cDNA was synthesized with random hexamer primers, followed by second strand cDNA synthesis using dTTP. Libraries then underwent end repair, A-tailing, adapter ligation, size selection, amplification, and purification. Sequencing was performed on the Illumina NovaSeq 6000 with 150bp paired-end reads. Reads were aligned to reference genome mm10 with Hisat2 v2.0.5, and raw read counts were determined using FeatureCounts v1.5.0-p3. Raw count normalization and differential expression analysis was performed using DESeq2 1.42.0^37^. Hierarchical clustering was performed for outlier detection (Extended Data Fig. 4A). Volcano plots were prepared using EnhancedVolcano 1.20 with apeglm fold change shrinkage^38^. For subsequent analysis, genes were considered significant if padj < 0.05.

### GSEA

Gene sets were ranked by t-stat determined by DESeq2 prior to enrichment analysis. GSEA and visualization was performed with clusterProfiler 4.11.0^39^. MitoCarta pathways were derived from a subset of mouse MitoCarta3.0 MitoPathways^40^, with the addition of GO:0006098 (pentose-phosphate shunt) and GO:0061621 (canonical glycolysis). Visualization of KEGG pathway “apoptosis” (mmu04210) was performed with pathview 1.42.0 and KEGGREST 1.42.0.

### Statistical analysis and visualization

Statistical significance was determined using GraphPad Prism v10 or R 4.3.2. All flow cytometry data were analyzed with FlowJo v10. Statistical tests used and n for each experiment are indicated in each figure legend. Sample sizes used are similar to our previous publication^4^. Experiments and data analysis were not performed blinded to genotype. Data distribution was assumed to be normal. Data was plotted and visualized with GraphPad Prism v10, or R packages clusterProfiler 4.11.0, ggplot2 3.5, pheatmap 1.0.12, or ComplexHeatmap 2.18.0^41^. Figures were prepared in Adobe Illustrator.

### Influenza Infection

Mouse adapted human influenza virus A/PR/8/34 (PR8) and A/X/31 (X31) and X31 were used for infection. Mice were exposed to aerosolized (Glas-Col) 500 TCID or PR8 in 7mL of saline. Details and time points of infection are outlined in the text. Expression of viral hemagglutinin (HA) in the lungs of infected mice was determined by real time PCR.

### BM Transfer

Bone marrow cells (CD45.2, 5 × 10^6^ cells) were injected intraorbitally into 8–12 week old irradiated B6 mice (CD45.1) (950 rad). Mice were then euthanized at time points as defined in the results.

### Flow Cytometry

Single-cell suspensions of tissues were prepared. Anti- CD4, CD8, CD44, B220, CD69, CD247, IgM, CXCR5, PD-1, Foxp3 antibody were purchased from BD Biosciences or Thermofisher scientific. Data were acquired on a Cytoflex S flow cytometer (Beckman Coulter) and analyzed using FlowJo software (Tree Star). Labeled tetramers (NIH tetramer core facility) were used to identify viral specific T cells. MitoTracker Green (Thermofisher), Total Reactive Oxygen Species (ROS) assay kit (Thermofisher) and TMRE (Abcam), 2-NBDG uptake kit (Abcam), MitoPY1 kit (Tocris Bioscience), ATP-Red (Millipore Sigma), were used according to manufacturer instructions. Apoptosis analysis was measured by Annexin V staining (ebioscience). Substrate cleavage by caspases were measured with caspase substrates PhiPhilus-G1D2, CaspaLux9- M2D2, CaspaLux8-L1D2 (OncoImmunin) according to the manufacturer instructions. Cells were loaded with 5 μM Cell Trace Violet (CTV) (ThermoFisher Scientific) and proliferation were estimated on day 3 by FACS. Gating strategy for CD4+ and CD8+ T cells is shown in Extended Data Fig 4B.

### Real-Time PCR

RNA was extracted from the tissues using Pure link RNA mini kit (Thermo Fisher Scientific) and was reverse transcribed to cDNA (iScript, Bio-Rad) according to the manufacturer’s instructions. Reactions were cycled and quantitated with an CFX96 Real Time Biorad PCR System (Applied Biosystems).

### In Vitro Differentiation

Naive (CD4^+^ (PerCP/Cy5.5, clone RM4–5) CD44^low^ (APC, clone IM7) CD62L^hi^ (eFluor 450, clone MEL-14) CD25^neg^ (PE, clone PC61.5) T cells) were purified by cell sorting. Purity was greater than 99% purity. Sorted naive CD4^+^ T cells (2 × 10^5^) were co-cultured at a ratio of 1:5 with mitomycin-treated T-depleted splenocytes as APCs in 48-well plates under various differentiation conditions for 3 days. Th1 conditions included 40 ng/ml IL-12 and anti-IL-4.Th17 used 20 ng/ml of IL-6, 5 ng/ml of TGF-β1, anti-IL-4, anti-IFN-γ, and anti-IL-12. Treg used 100 U/ml hIL-2, 5 ng/ml TGF-β1 with 1μg anti-CD3, and 10 μg/ml of each anti-IL-4, anti-IFN-γ, and anti-IL-12 antibodies. Antibodies were purchased from BioXcell unless otherwise indicated.

### OCR and ECAR Measurement

Oxygen consumption rate (OCR) and extracellular acidification rate (ECAR) were measured using a Seahorse XF^e^96 analyzer (Agilent). CD4^+^ or CD8^+^ T cells from mice activated for 24 hr with anti CD3 and anti CD28 were attached with Cell-Tak (Corning) according to manufacturer’s instructions at concentration 0.2 million cells/well in Seahorse BASE media with proprietary additives. Oxygen consumption rate (OCR) and extracellular acidification rate (ECAR) were determined using the Mitostress kit (Agilent) according to the manufacturer’s standard protocol. OCR and ECAR were calculated and recorded by the Seahorse XF^e^96 software. Complex IV activity (COX) was measured according to published methods^42^ using tetramethyl-p-phenylenediamine (TMPD) as an electron donor that is specific for complex IV with OCR as the readout.

### Immunization and Serum Analysis

Mice were immunized with 50 μg of TNP-CGG in Imject Alum (Pierce Chemical) and reimmunized with TNP-CGG alone in 28 days. Sera were tested by ELISA for TNP reactivity. Briefly, plates were coated with TNP-BSA or (10 μg/ml; Biosearch Technologies), and bound immunoglobulins were detected by alkaline phosphatase-conjugated detection antibodies to specific mouse isotypes (Southern Biotechnology Associates).

## Data Availability

All data, code, and materials used in the analysis will be made available via paper figures or supplements for purposes of reproducing or extending the analysis. RNAseq data is available at GEO under accession GSE269797.
